# A Colorimetric/Ratiometric Fluorescent Probe Based on Aggregation-Induced Emission Effect for Detecting Hypochlorous Acid in Real Samples and Bioimaging Applications

**DOI:** 10.3390/foods14142491

**Published:** 2025-07-16

**Authors:** Junliang Chen, Pingping Xiong, Huawei Niu, Weiwei Cao, Wenfen Zhang, Shusheng Zhang

**Affiliations:** 1 College of Food and Bioengineering, Henan University of Science and Technology, Luoyang 471000, China; chenjl2020@haust.edu.cn (J.C.); xpp18738636378@163.com (P.X.); caoweiwei@haust.edu.cn (W.C.); 2 Green Catalysis Center, College of Chemistry, Zhengzhou University, Zhengzhou 450001, China; zhangwenfen1988@126.com; 3 Food Laboratory of Zhongyuan, Luohe 462000, China

**Keywords:** small molecule fluorescent probe, hypochlorous acid, aggregation-induced emission, environmental monitoring, oxidative stress imaging, ferroptosis imaging

## Abstract

Hypochlorous acid (HClO) serves as a biological mediator and is widely utilized as a disinfectant in food processing and water treatment. However, excessive HClO residues in food and environmental water raise concerns due to the potential formation of carcinogenic chlorinated byproducts and disinfection byproducts (DBPs). Despite its importance, traditional methods for HClO detection often involve complex sample preparation, sophisticated instrumentation, and skilled operators. Herein, we report an aggregation-induced emission (AIE) small molecule fluorescent probe (**NYV**) that integrates colorimetric and ratiometric fluorescence responses for the detection of HClO. This probe exhibits high sensitivity, with a detection limit of 0.35 μM, a rapid response time of 1 min, and a wide linear range (0–142.5 μM), along with anti-interference capabilities, making it suitable for real-time monitoring. Furthermore, we have developed a portable solid-state sensor based on probe **NYV** for the rapid visual detection of HClO. The potential applications of this probe in real sample analysis and bioimaging experiments are demonstrated. Our findings contribute to the development of innovative fluorescent probes for HClO detection, with broad applications in food safety, environmental monitoring, and biomedical research on oxidative stress and ferroptosis.

## 1. Introduction

Hypochlorous acid (HClO), classified as a reactive oxygen species (ROS), plays a significant role in various physiological and pathological processes [1,2]. However, the excessive production of HClO is associated with tissue damage and a range of diseases, including atherosclerosis, neurodegenerative disorders, and cancer [3,4,5]. This chemical dichotomy highlights the urgent need for accurate detection of HClO across multiple fields. In food safety, the extensive use of HClO as a disinfectant in meat processing and vegetable washing necessitates rigorous monitoring of residual levels due to its potential to generate carcinogenic chlorinated byproducts [6]. Environmentally, HClO is a critical indicator of the efficacy of water disinfection [7]. However, the excessive application of HClO can lead to water pollution. When surplus HClO is released into natural water bodies, it interacts with organic matter present in the water, resulting in the formation of various disinfection byproducts (DBPs) [8]. These byproducts may pose significant risks to both human health and the environment [9,10]. Therefore, the dual role of HClO as a vital biological mediator and a potential health and environmental hazard highlights the urgent need for accurate detection methods and regulatory frameworks to balance its therapeutic uses with the management of toxic byproducts and ecological risks.

Traditional methods for the detection of HClO, including titration [11], chromatography [12], and electrochemical analysis [13], are often constrained by the complexities of sample preparation, the requirement for advanced instrumentation, and the necessity for skilled personnel. These limitations hinder their applicability in real-time and *on-site* monitoring contexts. In contrast, fluorescent probes have emerged as practical tools for HClO detection owing to their high sensitivity, selectivity, and user-friendly nature [14,15,16,17,18,19]. Despite these advancements, most existing HClO probes have severe limitations: (1) they rely on single emission intensity variations and are vulnerable to environmental disturbances and concentration fluctuations [20,21,22,23,24]; (2) aggregation-induced quenching (ACQ) effect, which limits their use in biological environments where probe aggregation is inevitable [25,26]; (3) slow response time or insufficient sensitivity to practical applications. Compared with single-emission sensors, dual-emission probes have significant advantages, including improved detection accuracy, reduced environmental interference, and enhanced reliability, making them highly suitable for real-time and on-site monitoring applications [27].

To address these challenges, we have developed probe **NYV**, an AIE-active dual-mode probe that integrates colorimetric and ratiometric fluorescence responses. The design strategy employs a coumarin–pyrene hybrid architecture and diphenylphosphine groups responsive to HClO. Upon interaction with HClO, the probe facilitates ratiometric measurements (I_460_/I_575_) accompanied by a significant fluorescence emission shift (115 nm), effectively mitigating signal drift. The AIE properties of the probe ensure enhanced emission in aggregated states, which is essential for maintaining sensitivity in biological environments. Notably, probe **NYV** achieves a detection limit of 45 nM with a response time of 1 min, surpassing the performance of most existing probes.

## 2. Experimental Section

### 2.1. Materials and Chemicals

4-(Diethylamino) salicylaldehyde, ethyl acetoacetate, phosphorus oxychloride, and 1-pyrenecarboxaldehyde were sourced from Shanghai Titan Scientific Co., Ltd. (Shanghai, China) Deionized water (18.25 MΩ·cm) was utilized throughout the experimental procedures. Tap water was collected from Building No. 4 of the Engineering Department at Henan University of Science and Technology. Environmental water samples were gathered from the Luo and Yi Rivers, which traverse Luoyang. The samples were collected at a depth of 20–30 cm below the water surface to ensure representative sampling, stored in sterile polyethylene bottles, and transported to the laboratory within 1 h. Before analysis, the sample was filtered through a 0.22 μm filter membrane to remove insoluble particles. The filtered samples were stored in the refrigerator at 4 °C and used within 24 h of collection to ensure sample integrity.

### 2.2. Instruments and Equipment

A Bruker Esquire 3000 plus mass spectrometer was employed for mass spectral analysis. NMR spectra were recorded using a Bruker DTX-400 spectrometer (Bruker, Billerica, MA, USA). pH values were determined with a pH-3C meter. UV-vis spectra were acquired using a UV-2600 UV/Vis spectrometer. Fluorescence spectra were obtained with an Agilent Cary Eclipse 9800A fluorescence spectrophotometer (Agilent, Santa Clara, CA, USA).

### 2.3. Synthesis of Probe NYV

Compound **1** was synthesized using the existing literature [28]. Specifically, Compound **1** (518.24 mg, 2 mmol) and pyrene-1-carboxaldehyde (460.14 mg, 2 mmol) were dissolved in absolute ethanol (40 mL), and 100 μL of piperidine was added as a catalyst. The reaction mixture was condensed and refluxed at 80 °C for 14 h, and the process was monitored by thin-layer chromatography (TLC, Rf = 0.37) using a silica gel plate (GF254) with a mobile phase of dichloromethane (CH_2_Cl_2_). The TLC plates were visualized under UV light (365 nm). Upon completion of the reaction, the solution was subjected to suction filtration to isolate an orange solid. The crude product was purified via silica gel column chromatography using CH_2_Cl_2_ as the eluent, yielding probe **NYV** (289 mg, 0.61 mmol). Yield: 30.5%. ^1^H NMR (400 MHz, CDCl_3_) δ 8.99 (d, *J* = 15.6 Hz, 1H), 8.66–8.56 (m, 2H), 8.53 (d, *J* = 8.4 Hz, 1H), 8.44 (d, *J* = 15.6 Hz, 1H), 8.24–8.13 (m, 4H), 8.11–7.98 (m, 3H), 7.38 (d, *J* = 8.8 Hz, 1H), 6.56 (dd, *J* = 8.8, 2.0 Hz, 1H), 6.45 (d, *J* = 2.0 Hz, 1H), 3.41 (q, *J* = 7.2 Hz, 4H), 1.22 (t, *J* = 7.2 Hz, 6H). ^13^C NMR (101 MHz, CDCl_3_) δ 186.3, 161.0, 158.6, 152.9, 148.79, 139.6, 132.7, 131.7, 131.3, 130.7, 130.3, 129.2, 128.4, 128.3, 127.4, 126.8, 126.1, 125.8, 125.7, 125.1, 124.9, 124.8, 124.6, 122.8, 116.7, 109.8, 108.7, 96.6, 45.1, 12.4. HR-MS: Calcd for [C_32_H_27_NO_3_ + H]^+^: 472.1907; Found 472.1905.

### 2.4. Real Samples Preparation

Before analysis, real samples of tap water, Luo River water, and Yi River water were subjected to microfiltration using a 0.22 μm polyethersulfone (PES) membrane filter to remove insoluble particles and ensure sample clarity. Each sample was reacted with probe **NYV** (10 μM) for 1 min, after which the fluorescence intensity was measured. Additionally, spike recovery testing necessitated the precise addition of HClO at concentrations of 0, 30, 60, and 90 μM. The resulting fluorescence intensity was detected and recorded at 460 and 575 nm with an excitation wavelength of 400 nm. Each experiment was repeated 3 times. During fluorescence detection, the sample was excited at 400 nm, and the emission intensities were recorded at 420–700 nm using the Agilent Cary Eclipse 9800A fluorescence spectrophotometer. The slit widths for excitation and emission are set at 10 nm and 5 nm, respectively, and the voltage of the photomultiplier tube (PMT) is maintained at 700 V to ensure consistent sensitivity. The fluorescence intensity ratio (I_460_/I_575_) was calculated to quantify the concentration of HClO, as this ratio method minimizes environmental interference and improves the reliability of detection.

### 2.5. Preparation of the Portable Solid Sensor

Filter paper was immersed in 50 μM probe **NYV** for 10 min, and then dried after staining. Subsequently, the prepared test paper was exposed to various concentrations of aqueous HClO solution for 20 s. After reacting at room temperature for 1 min, colorimetric responses were observed visually, and ratiometric responses were recorded.

### 2.6. Calculation of Detection Limits and Quantification Limits

Using the relationships of “detection limit = 3 *σ*/K and quantification limit = 10 *σ*/K (*σ* is the standard deviation of blank measurement, K is the slope of calibration curve)”, we studied the linear correlation between HClO concentration and fluorescence intensity.

### 2.7. Cytotoxicity of Probe NYV

The cytotoxic effects of probe **NYV** on HeLa cells were evaluated using a CCK-8 assay. Cells were seeded in 96-well plates (2 × 10^4^ cells/well; 100 μL complete medium per well) and cultured overnight (37 °C, 5% CO_2_). Upon reaching 70–80% confluence, the medium was aspirated, and cells were gently washed with PBS. Fresh medium containing probe **NYV** at graded concentrations (0, 2.5, 5, 10, 15, 20, and 30 μM) was added, followed by 24 h incubation. Subsequently, the medium was replaced with 10% CCK-8 solution and incubated for 1 h. Absorbance at 450 nm was measured using a microplate reader (triplicate wells per concentration).

### 2.8. Preparation of the Cell Imaging Experiments

HeLa cells were cultured in Dulbecco’s Modified Eagle Medium (DMEM) supplemented with 10% fetal bovine serum (FBS, 37 °C, 5% CO_2_).

#### 2.8.1. Confocal Imaging Experiments of Probe NYV

(1) HeLa cells were incubated with probe **NYV** (5 µM, 1 h); (2) HeLa cells were treated with HClO (200 µM, 1 h), followed by incubation with probe **NYV** (5 µM, 1 h); (3) HeLa cells were incubated with Pb^2+^ (100 µM, 1 h), and then probe **NYV** (5 µM, 1 h) was added. Conditions: λ_ex_ = 405 nm. Scale bar = 50 μm.

#### 2.8.2. LPS-Induced Confocal Imaging Experiment on Oxidative Stress

(1) HeLa cells were incubated with probe **NYV** (5 μM, 1 h); (2) HeLa cells were treated with LPS (2 μg/mL) and PMA (1 μg/mL) for 24 h, followed by incubation with probe **NYV** (5 μM, 1 h); (3) HeLa cells were incubated with LPS (2 μg/mL) and PMA (1 μg/mL) for 24 h, then incubated with NAC (1 mM, 1 h), and finally incubated with probe **NYV** (5 μM, 1 h).

#### 2.8.3. Changes in HClO Levels in Cells During Ferroptosishe

(1) HeLa cells were incubated with probe **NYV** (5 μM, 1 h); (2) HeLa cells were treated with Erastin (10 μM, 24 h), followed by incubation with probe **NYV** (5 μM, 1 h); (3) HeLa cells were incubated with both Erastin (10 μM) and GSH (1 mM) for 24 h, followed by incubation with probe **NYV** (5 μM, 1 h).

## 3. Results and Discussion

**Scheme 1:**
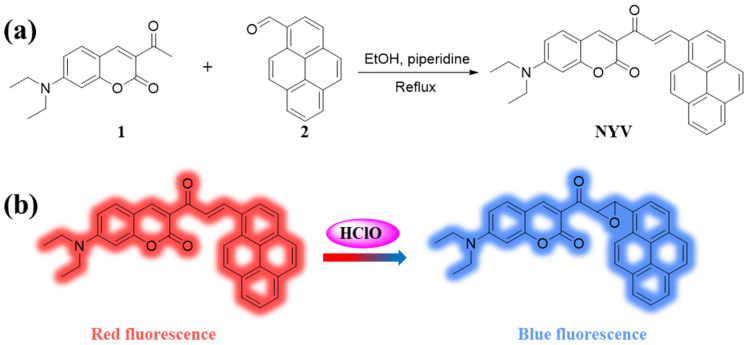
(**a**) Synthetic route of probe **NYV**. (**b**) Possible recognition mechanism of HClO by probe **NYV**.

In the design of probe **NYV**, coumarin and pyrene fluorophores were employed for strategic integration. Coumarin, a widely utilized fluorescent dye, is recognized for its exceptional photostability and high fluorescent quantum yield [29]. In contrast, pyrene is distinguished by its unique fluorescent characteristics and substantial Stokes shift, effectively reducing self-absorption and enhancing detection accuracy [30,31,32]. Furthermore, pyrene is classified as a typical AIE fluorophore [33,34]. This property renders AIE-based fluorescent probes particularly effective for the detection of complex samples, including food, environmental water samples, and biological materials. Upon exposure to HClO, a significant alteration in the solution color of probe **NYV** is observed, providing a straightforward and intuitive visual indicator during the detection process. Simultaneously, the fluorescence intensity of the probe experiences a ratiometric transformation. This dual-mode approach, which combines colorimetric and ratiometric fluorescence techniques, enhances detection accuracy and reliability, effectively mitigating the impact of external environmental interferences. The synthesis of probe **NYV** was accomplished through a one-step process, with the detailed methodology delineated in Scheme 1. The chemical structure of probe **NYV** was rigorously confirmed through ^1^H NMR, ^13^C NMR, and HR-MS analyses, as illustrated in Figure S1.

### 3.1. Spectral Response of Probe NYV to HClO

**Figure 1:**
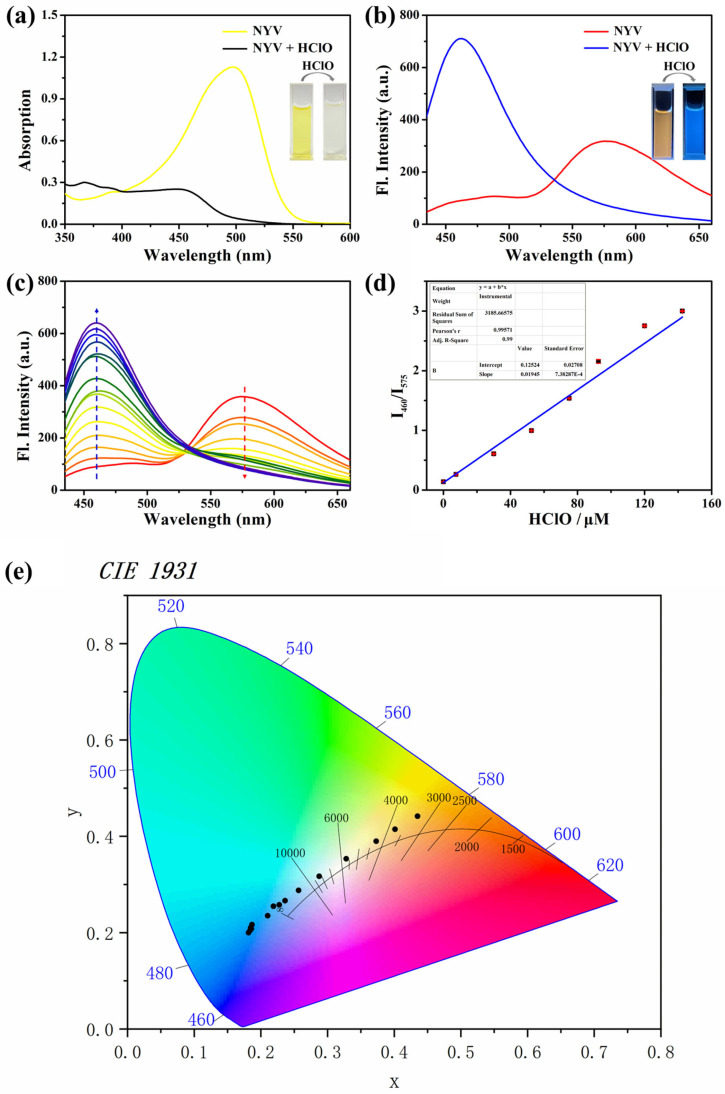
(**a**) Absorption and (**b**) fluorescence spectra of probe **NYV** (10 μM) before and after HClO (300 μM) treatment. (**c**) Fluorescence response of probe **NYV** (10 μM) to increasing HClO concentrations (0–300 μM). (**d**) A linear correlation between the emission intensity ratio (I_460_/I_575_) of probe **NYV** (10 μM) and HClO concentrations (0–142.5 μM). (**e**) The CIE 1931 color diagram of HClO titration. Conditions: λ_ex_ = 400 nm, slits = 10 nm/5 nm.

To investigate the recognition capability of probe **NYV** towards hypochlorous acid (HClO), we comprehensively analyzed its UV-Vis and fluorescence spectra. Figure 1a,b illustrate the spectral changes of probe **NYV** before and after HClO recognition. The probe shows a significant decrease in absorption at 500 nm and a reduction in fluorescence intensity at 575 nm, while intensity increases at 460 nm, indicating a ratiometric change that enhances HClO detection accuracy. The emission spectra of the free probe exhibit the unusual characteristics of two distinct emission peaks. This phenomenon may be related to the molecular structure of the probe, which contains a chromophore with two different emission centers. The relative rotation between coumarin and pyrene groups may affect the intramolecular energy transfer and emission processes. Moreover, the probe exhibits an AIE effect, which may cause the fluorescent probe to aggregate, potentially affecting intramolecular energy transfer and emission processes. Although the exact mechanism of this abnormal emission requires further research, it is worth noting that similar phenomena have already been reported in the literature [35,36,37,38,39,40,41]. As the concentration of HClO increases, the color of the probe solution changes from yellow to colorless (Figure 1a), and the fluorescence changes from orange-yellow to blue (Figure 1b). This dual change confirms the effective recognition capability of probe **NYV** for HClO and provides a simple detection method. A good linear relationship exists between the fluorescence intensity of probe **NYV** and the concentration of HClO, particularly from 0 to 142.5 μM, with a detection limit of 0.35 μM (Figure 1c,d and Table S1). These results show that probe **NYV** is highly sensitive and accurately detects HClO at low concentrations, making it an efficient and intuitive tool for HClO recognition with significant research value and broad application potential.

### 3.2. Selectivity and Anti-Interference Detection of Probe NYV for HClO

**Figure 2:**
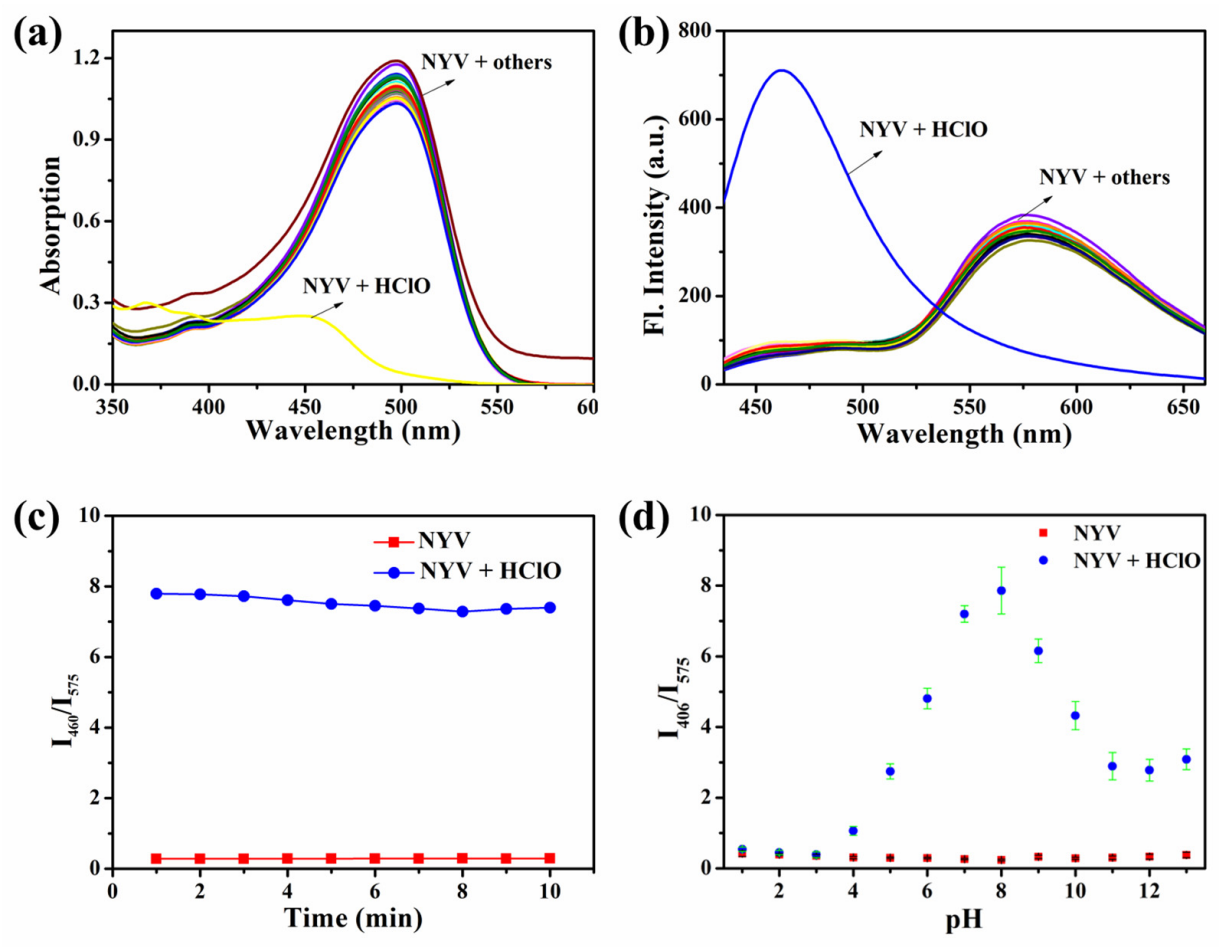
(**a**) Variations in absorption and (**b**) fluorescence intensity of probe **NYV** (10 μM) upon treatment with HClO (300 μM) and various analytes (300 μM of Na^+^, K^+^, F^−^, Cl^−^, Br^−^, I^−^, CO_3_^2−^, HCO_3_^−^, Ac^−^, SO_4_^2−^, HSO_3_^−^, NO_3_^−^, NO_2_^−^, HS^−^, Cys, Hcy, GSH, NO, H_2_O_2_, ^1^O_2_). (**c**) Time-dependent fluorescence response of probe **NYV** (10 µM) to HClO (300 µM). (**d**) Fluorescence intensity ratio (I_460_/I_575_) at different pH values. Conditions: λ_ex_ = 400 nm, λ_em_ = 460 nm/575 nm, slits = 10 nm/5 nm.

Due to the presence of various ions and biomolecules in food and biological samples, these components may interfere with detection processes. Consequently, an effective probe should accurately identify and respond to target molecules in these complex environments. As illustrated in Figure 2a,b, the addition of a wide range of common interfering substances (Na^+^, K^+^, F^−^, Cl^−^, Br^−^, I^−^, CO_3_^2−^, HCO_3_^−^, Ac^−^, SO_4_^2−^, HSO_3_^−^, NO_3_^−^, NO_2_^−^, HS^−^, Cys, Hcy, GSH, NO, H_2_O_2_, ^1^O_2_) resulted in negligible changes to the UV–visible absorption and fluorescence emission spectra of probe **NYV**. This demonstrates that probe **NYV** exhibits exceptional selectivity for HClO, maintaining a specific response even in the presence of these substances. When HClO was introduced alongside these substances, the fluorescence intensity remained nearly identical to that observed with HClO alone (Figure S2). Thus, probe **NYV** can accurately identify HClO without significant interference, showcasing high selectivity and remarkable anti-interference capabilities. These features make probe **NYV** a promising candidate for food and biological detection applications, enabling reliable quantification of HClO levels and supporting related research.

### 3.3. Time-Dependent and the Effects of pH Responses of NYV Towards HClO

The rapid response capability and broad pH applicability of probes are essential characteristics that significantly enhance the efficiency and accuracy of detection processes. These attributes also provide a solid foundation for its diverse applications across multiple fields. The experimental data illustrated in Figure 1c support this assertion, indicating that probe **NYV** achieves a response plateau within 1 min, a critical metric in evaluating probe performance that highlights its exceptional response speed. The pH value, a crucial factor influencing biochemical reactions and the states of materials, substantially impacts detection outcomes. Figure 1d demonstrates that the probe maintains stable fluorescence performance across a pH range of 5 to 12, thereby greatly expanding its potential applications. Consequently, the probe’s advantages of rapid response and extensive pH applicability suggest that this probe holds considerable potential and value in various domains.

### 3.4. AIE Characterization of Probe NYV

To further verify the AIE characteristics of probe **NYV**, we conducted fluorescence spectroscopy measurements of the probe in both solution state and aggregated state. The behavior of AIE was evaluated by comparing the fluorescence emission spectra of probe **NYV** in a dilute solution (good solvent, acetonitrile) and its aggregation state (poor solvent, acetonitrile/water mixture, with an increase in the water component). Figure S3 shows the fluorescence emission spectra of probe **NYV** in solution and aggregated states. In dilute acetonitrile solution, due to molecular rotation and non-radiative decay pathways, the fluorescence emission of probe **NYV** is relatively weak. However, as the water component in the acetonitrile/water mixture exceeds 60%, the fluorescence intensity of probe **NYV** significantly increases. This enhancement of fluorescence intensity is a characteristic of the AIE active fluorophore, confirming the AIE behavior of probe **NYV**.

### 3.5. Solid-State Sensors for HClO Detection

**Figure 3:**
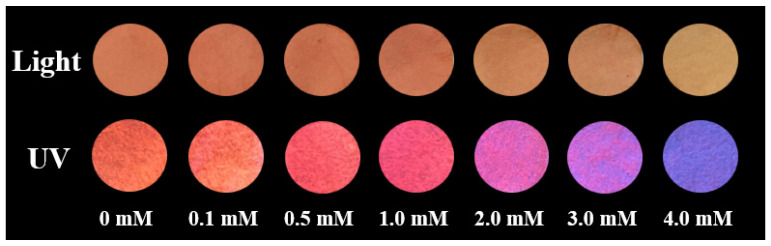
Filter papers soaked in HClO concentrations (0, 0.1, 0.5, 1.0, 2.0, 3.0, and 5.0 mM) were exposed to white light (**top**) and UV (**bottom**) light.

In recent years, advancements in solid sensor technology that employ colorimetric changes have significantly improved the rapid detection of target substances [42,43]. As the concentration of HClO increases, the color of the solid sensor transitions progressively from light brown to yellow when exposed to white light (Figure 3, top). Furthermore, under UV illumination (365 nm), the sensor gradually shifts from orange to blue (Figure 3, bottom), indicating variations in HClO concentration. This colorimetric response offers an intuitive representation of the increasing HClO levels, thereby facilitating a visual assessment of its concentration. Through its distinctive color response mechanism, this innovative solid sensor enables immediate visual detection of HClO concentration, streamlining the detection process and enhancing overall detection efficiency.

### 3.6. Detection of HClO in Various Real Samples

To evaluate the practical utility of probe **NYV** in real-world contexts, a series of experiments were conducted to detect HClO in different environmental samples, such as tap water, Luo River water, and Yi River water. Each sample was treated with probe **NYV** for 1 min, after which fluorescence intensity was measured. Spike recovery tests were performed to assess method accuracy by supplementing samples with HClO at 0 μM, 30 μM, and 60 μM concentrations. The fluorescence intensities were subsequently measured and recorded at wavelengths of 460 nm and 575 nm to ascertain the HClO concentration in each sample. The results of these experiments, as detailed in Table 1, indicate that the recovery rates of HClO range from 83.21% to 107.17%, with relative standard deviations (RSD) consistently below 4.40%. The probe’s ability to accurately quantify HClO in diverse real samples highlights its potential for practical applications in environmental monitoring. Moreover, the simplicity and rapidity of the detection method make it suitable for large-scale screening and continuous monitoring of HClO levels in environmental water samples.

### 3.7. Bioimaging of Probe NYV for Detecting HClO

**Figure 4:**
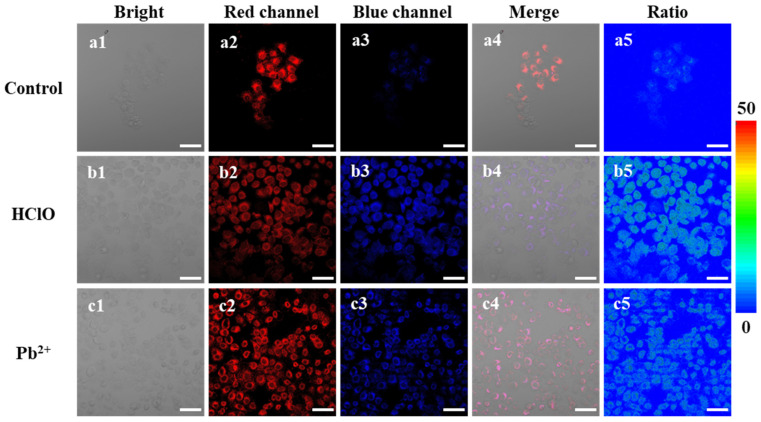
Confocal imaging of HeLa cells using probe **NYV**. (**a1**–**a5**) Cells were treated with probe **NYV** (5 µM, 1 h). (**b1**–**b5**) Cells were exposed to HClO (200 µM, 1 h) followed by probe **NYV** (5 µM, 1 h). (**c1**–**c5**) Cells were exposed to Pb^2+^ (100 µM, 1 h) followed by probe **NYV** (5 µM, 1 h). Conditions: λ_ex_ = 405 nm. Scale bar = 50 μm.

Building on the probe **NYV**’s advantageous spectral characteristics, we initiated efforts to expand its application scope, particularly emphasizing its potential for detecting HClO within biological systems. Initially, we utilized the CCK-8 assay to investigate the cytotoxicity of probe **NYV** on HeLa cells. As illustrated in Figure S4, cell viability was evaluated after incubating with probe **NYV** (0, 2.5, 5, 10, 15, 20, and 30 μM) for 24 h. At the highest concentration tested (30 μM), cell viability remained above 85%, indicating that probe **NYV** exhibits relatively favorable biocompatibility and low cytotoxicity. Subsequently, we conducted experiments to explore the ability of probe **NYV** to detect exogenous HClO within cellular environments. As depicted in Figure 4(b2,b3), the addition of HClO decreased red channel fluorescence and significantly increased blue channel fluorescence. This alteration indicates that probe **NYV** can effectively recognize HClO in cellular contexts, underscoring its distinctive advantages in the detection of HClO.

### 3.8. Changes in HClO Levels in Cells During Oxidative Stress

Oxidative stress is a significant factor in the pathogenesis of various diseases (Forman & Zhang), thereby necessitating the investigation of its underlying mechanisms to develop novel therapeutic strategies. Given the health risks associated with heavy metal ions, particularly lead ions (Pb^2+^), and their potential to disrupt physiological functions through the modulation of oxidative stress, we examined the relationship between Pb^2+^ and the generation of HClO. Our findings revealed that Pb^2+^ reduced fluorescence intensity in the red channel while increasing intensity in the blue channel (Figure 4(c2,c3), analogous to those induced by HClO. This suggests that Pb^2+^ may enhance the production of HClO. These results strengthen our comprehension of the impact of heavy metal ions on cellular damage and oxidative stress, thereby opening new avenues for research into the applications of probe **NYV**.

**Figure 5:**
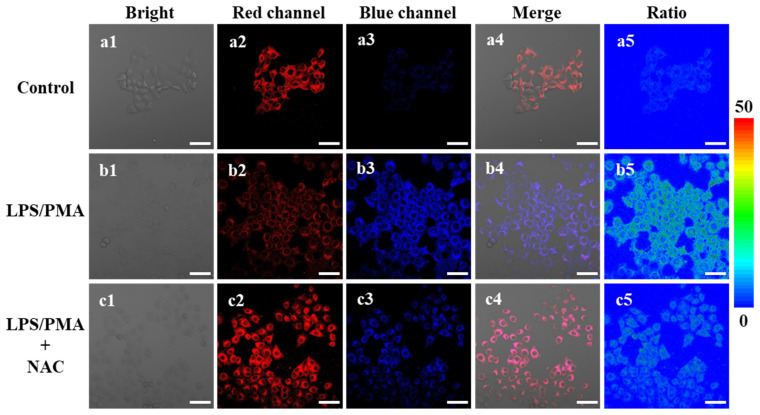
Confocal imaging of LPS/PMA-induced oxidative stress in HeLa cells using probe **NYV**. (**a1**–**a5**) Cells were incubated with probe **NYV** (5 μM, 1 h). (**b1**–**b5**) Cells were treated with LPS (2 μg/mL) and PMA (1 μg/mL) for 24 h, followed by incubation with probe **NYV** (5 μM, 1 h). (**c1**–**c5**) Cells were exposed to LPS (2 μg/mL) and PMA (1 μg/mL) for 24 h, subsequently incubated with NAC (1 mM, 1 h), and finally incubated with probe **NYV** (5 μM, 1 h). Conditions: λex = 405 nm. Scale bar = 50 μm.

Building on this understanding, we further investigated the effects of lipopolysaccharide (LPS, a robust inducer of inflammatory responses) and phorbol 12-myristate 13-acetate (PMA, an oxidative stress activator) on the oxidative stress status of HeLa cells, as well as the interventional efficacy of the antioxidant N-acetylcysteine (NAC, a ROS scavenger) [44,45,46]. The results in Figure 5(b2,b3) reveal a marked increase in blue fluorescent signal within the cells and a reduction in red fluorescence. This observation suggests that treatment with LPS and PMA significantly elevated intracellular HClO levels. In contrast, the blue fluorescent signal was markedly reduced in the treatment group with NAC added (Figure 5(c3)), proving that NAC can effectively neutralize intracellular HClO. The findings demonstrate that LPS and PMA can significantly induce oxidative stress responses in HeLa cells, while NAC, as a highly effective antioxidant, can effectively inhibit such stress responses. This discovery not only deepens our understanding of the mechanisms of oxidative stress but also provides robust support for the development of antioxidant therapeutic strategies.

### 3.9. Changes in HClO Levels in Cells During Ferroptosis

**Figure 6:**
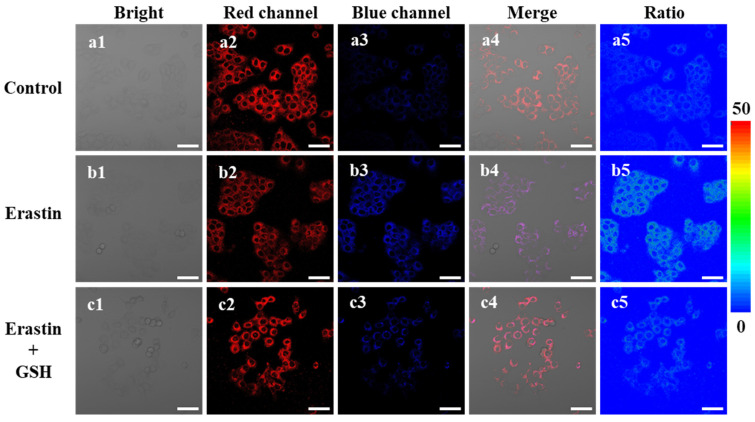
Confocal imaging of ferroptosis in HeLa cells using probe **NYV**. (**a1**–**a5**) Cells were exposed to probe **NYV** for 1 h. (**b1**–**b5**) Cells were exposed to Erastin (10 Μm, 24 h), and subsequently incubated with probe **NYV** (5 μM, 1 h). (**c1**–**c5**) Cells were exposed to both Erastin (10 μM) and GSH (1 mM) for 24 h, and subsequently incubated with probe **NYV** (5 μM, 1 h). Conditions: λ_ex_ = 561 nm, λ_em_ = 625–665 nm. Scale bar = 50 μm.

Ferroptosis, a recently characterized form of programmed cell death, has garnered considerable attention in the scientific community [47,48]. Erastin, a well-established inducer of ferroptosis, has been the subject of extensive investigation regarding its mechanism of action and the intracellular alterations it induces [49]. Our experimental findings revealed that treatment with Erastin significantly increased blue channel fluorescence (Figure 6(b3)) and decreased red channel fluorescence (Figure 6(b2)). This observation suggests a substantial production of HClO within the cells. The increased generation of HClO is likely associated with elevated oxidative stress during ferroptosis. To further investigate this phenomenon, we introduced GSH, a critical antioxidant, in subsequent experiments. The results indicated that GSH treatment enhanced red channel fluorescence while concurrently reducing blue channel fluorescence (Figure 6(c2,c3)), demonstrating that GSH effectively mitigates HClO levels induced by Erastin. This finding implies that GSH intervention may play a regulatory role in HClO production during ferroptosis, potentially influencing its progression and providing new avenues for therapeutic targets in the treatment of ferroptosis-related diseases.

## 4. Conclusions

This study successfully developed a small molecule fluorescent probe, **NYV**, for sensitive and selective HClO detection. The probe integrates the benefits of colorimetric and ratiometric fluorescence, offering a simple and intuitive visual cue and enhanced detection accuracy and reliability. The results indicate that probe **NYV** has high sensitivity, rapid response, and excellent selectivity, even in various interfering substances. Its performance in detecting HClO in environmental water was verified, showcasing its broad application potential. Furthermore, the probe’s utility in bioimaging experiments demonstrates its capability to visualize intracellular HClO concentrations, providing valuable insights into oxidative stress and ferroptosis-related research. This study contributes to developing novel fluorescent probes for HClO detection, with significant implications for environmental monitoring and biomedical research.

## Figures and Tables

**Table 1:** The recovery rate of real samples.

Sample	Spiked (μM)	Found (μM)	Recover (%)	RSD (n = 3, %)
Tap water	0	1.37 ± 0.06	–	–
	30.0	28.62 ± 0.26	95.30	0.85
	60.0	62.19 ± 1.17	103.61	0.76
Luohe water	0	Not detected	–	–
	30.0	29.97 ± 0.44	99.91	1.48
	60.0	51.84 ± 2.18	86.41	4.20
Yihe water	0	Not detected	–	–
	30.0	24.96 ± 1.10	83.21	4.40
	60.0	64.30 ± 1.32	107.17	2.05

## Data Availability

The original contributions presented in the study are included in the article, further inquiries can be directed to the corresponding authors.

## Supplementary Materials

The following supporting information can be downloaded at https://www.mdpi.com/article/10.3390/foods14142491/s1, Figure S1: Structure characterization of probe **NYV**; Figure S2: Fluorescence intensity changes of probe **NYV** after treatment with HClO and other respective species; Figure S3: AIE characterization of probe **NYV**; Figure S4: Cytotoxicity of probe **NYV**; Table S1: Standard deviation (σ) of blank measurement.
