# Supplementary material for: A Colorimetric/Ratiometric Fluorescent Probe Based on Aggregation-Induced Emission Effect for Detecting Hypochlorous Acid in Real Samples and Bioimaging Applications
Source: Foods. 2025 Jul 16;14(14):2491. doi: 10.3390/foods14142491 (PMC12294225; doi:10.3390/foods14142491)
Supplement: Supplementary file 1 [file foods-14-02491-s001.zip › foods-3695916-supplementary.pdf]

Electronic Supplementary Information

A colorimetric/ratiometric dual-mode fluorescent probe based on aggregation-induced emission effect for detecting hypochlorous acid in real samples and bioimaging applications

Junling Chen[a], Pingping Xiong[a], Huawei Niu[a,b,*], Weiwei Cao[a,*], Wenfen Zhang[b,c], Shusheng Zhang[b,c,*]

[a]*College of Food and Bioengineering, Henan University of Science and Technology, Luoyang, 471000, P. R. China*

[b]*Green Catalysis Center, and College of Chemistry, Zhengzhou University, Zhengzhou, 450001, PR China*

[c]*Food Laboratory of Zhongyuan, Luohe, 462000, PR China*

**Corresponding author; Email: niuhw0816@126.com (H. W. Niu); caoweiwei@haust.edu.cn (W. W. Cao); zsszz@126.com (S. S. Zhang).*

Table of contents

Figure S1 Structure characterization of probe **NYV**

Table S1 Standard deviation (σ) of blank measurement

Figure S2 Fluorescence intensity changes of probe **NYV** after treatment with HClO and other respective species

Figure S3 AIE characterization of probe **NYV**

Figure S4 Cytotoxicity of probe **NYV**

Figure S1 Structure characterization of probe **NYV**

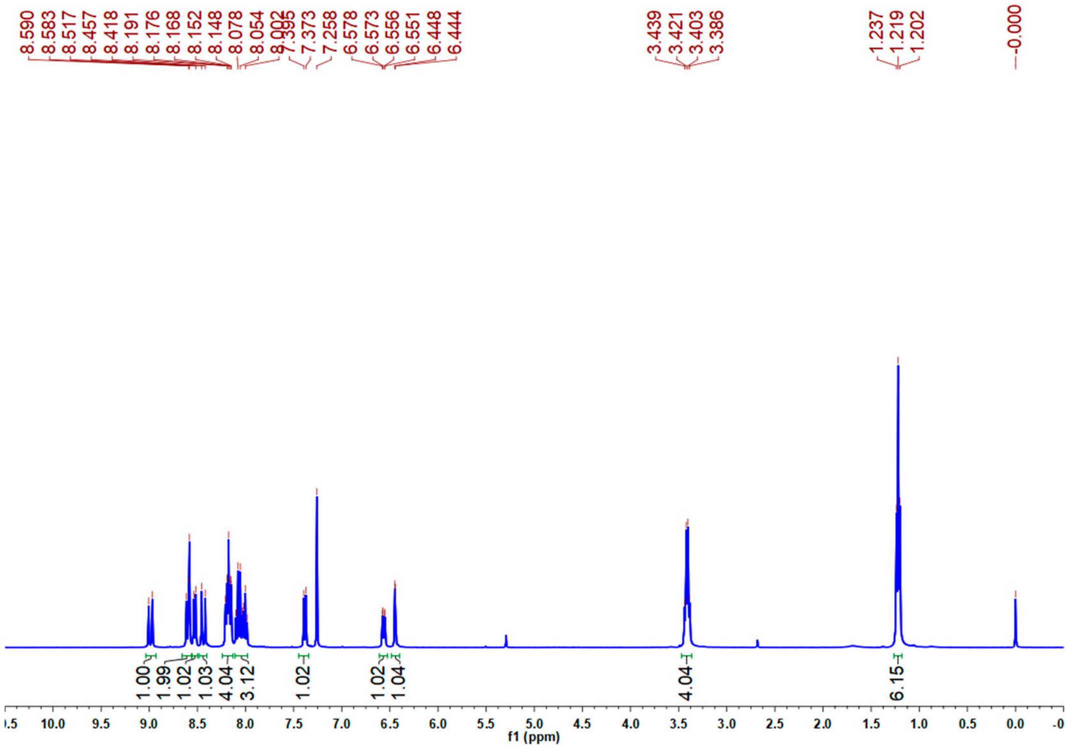

^{1}H-NMR spectrum of probe **NYV** in $CDCl_3$

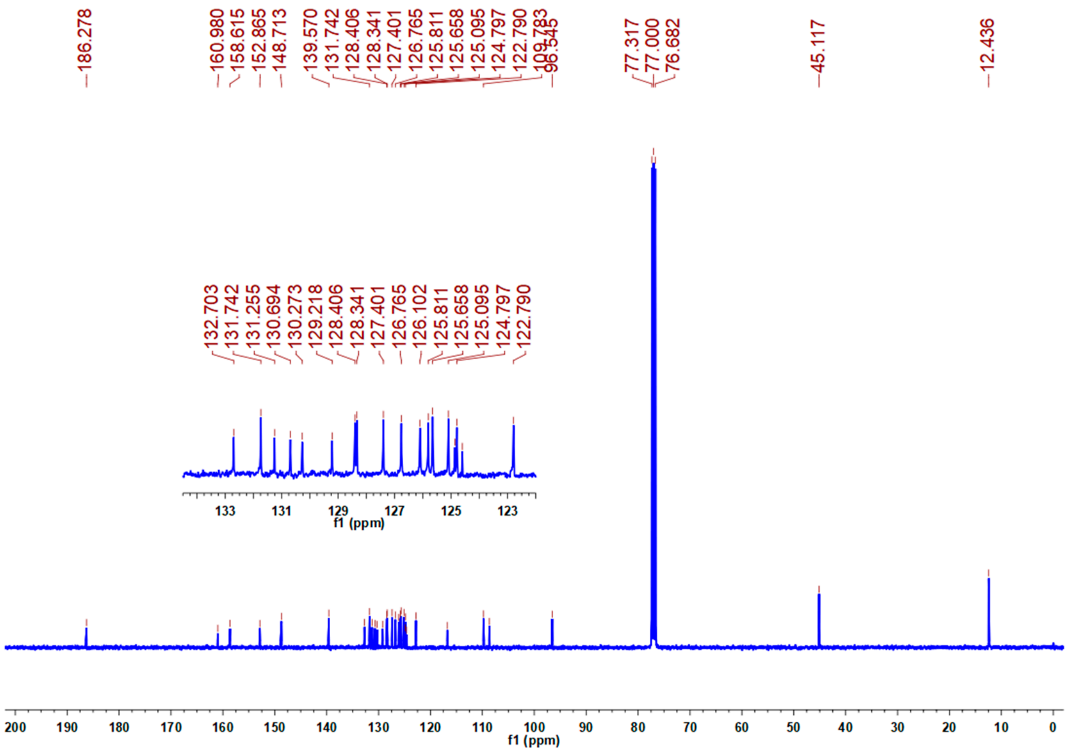

^{13}C-NMR spectrum of probe **NYV** in $CDCl_3$

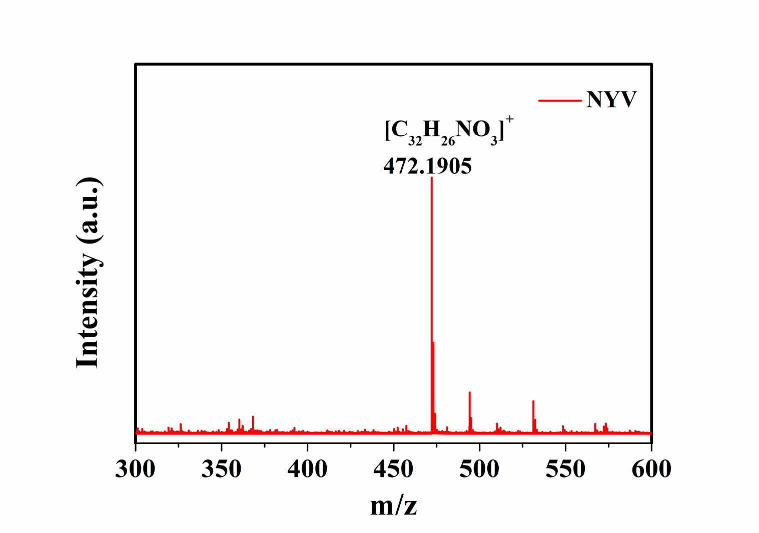

HR-MS spectrum of probe **NYV** (in MeOH)

Table S1 Standard deviation (σ) of blank measurement.

λ_{em} = 491 nm	
0.283508307	
0.287140562	
0.285834718	
0.281738455	
0.284604217	
0.282128619	
0.282515318	
0.283015688	
0.287260818	
0.281058761	
0.286263623	
0.283376401	
0.287032085	
0.287784619	
0.285578237	
σ	0.002255472

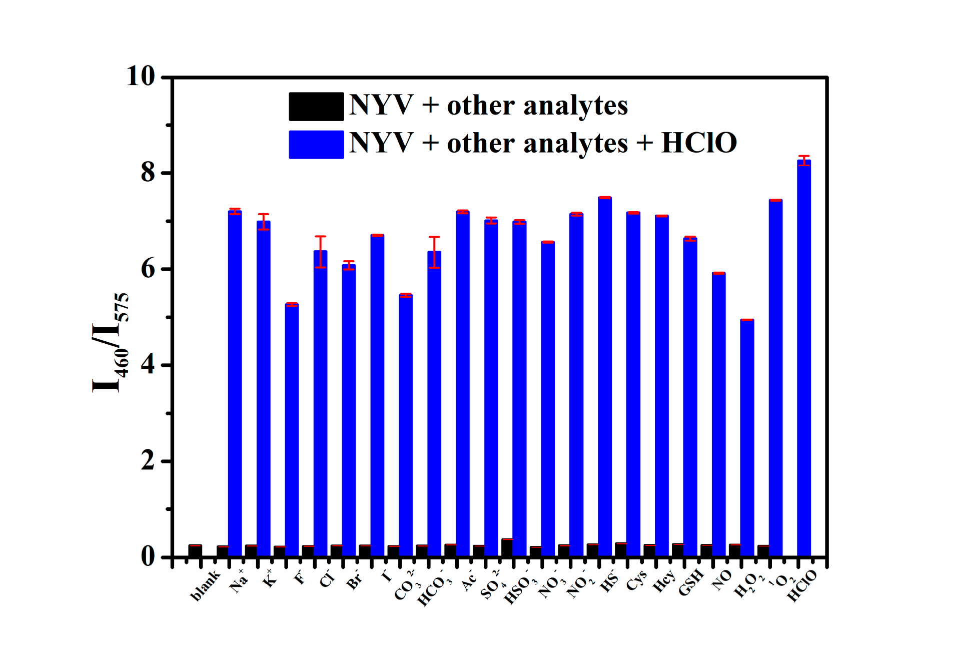

Figure S2 Fluorescence intensity changes of **NYV** (10 μM) after treatment with HClO (300 μM) and other respective species (300 μM of Na^+, K^+, F^-, Cl^-, Br^-, I^-, CO_3^{2-}, HCO_3^-, Ac^-, SO_4^{2-}, HSO_3^-, NO_3^-, NO_2^-, HS^-, Cys, Hcy, GSH, NO, H_2O_2, $^1O_2$).

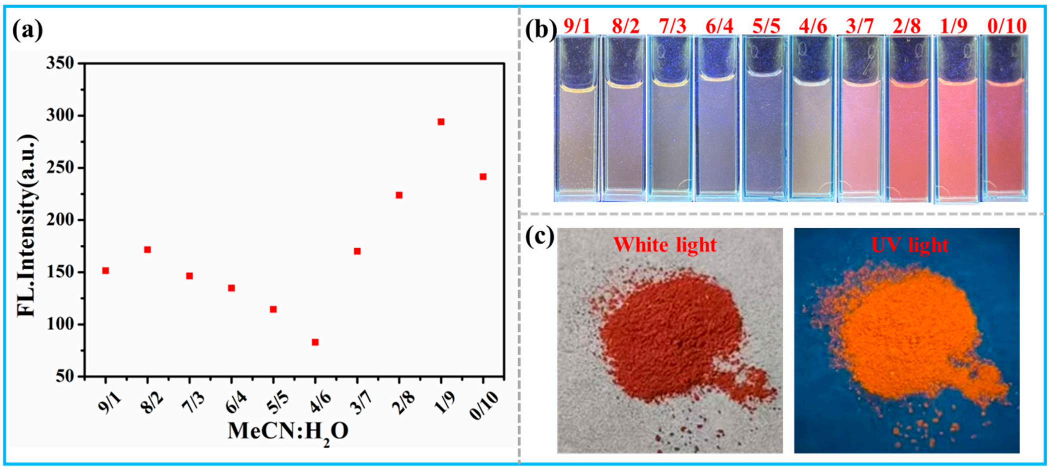

Figure S3 Fluorescence emission spectra of probe **NYV** (10 μM) in MeCN (solution state) and MeCN/water mixtures with increasing water fractions (aggregated state). Conditions: λ_{ex} = 400 nm, slits = 10 nm/5 nm.

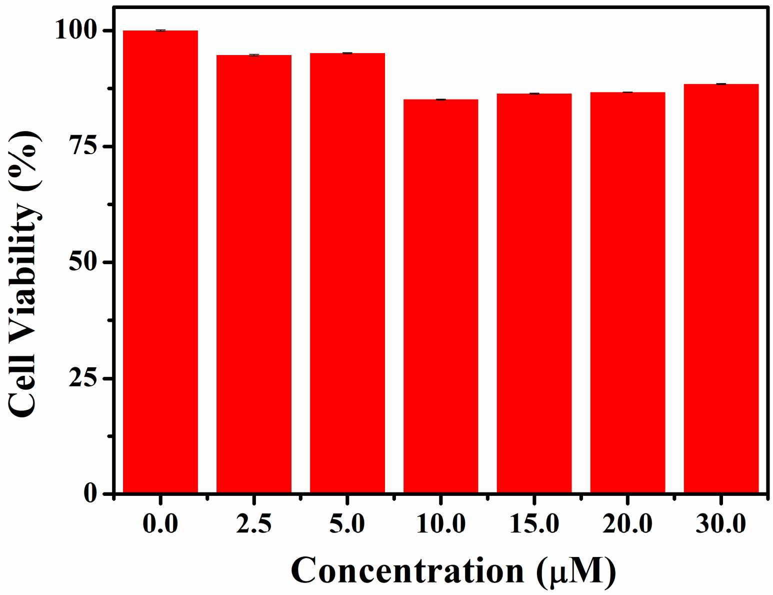

Figure S4 Cytotoxicity of probe **NYV** with different concentrations (μM).
